# Natural Intermediate Band in I_**2**_-II-IV-VI_4_ Quaternary Chalcogenide Semiconductors

**DOI:** 10.1038/s41598-018-19935-5

**Published:** 2018-01-25

**Authors:** Qiheng Liu, Zenghua Cai, Dan Han, Shiyou Chen

**Affiliations:** 1 Key Laboratory of Polar Materials and Devices (MOE), East China Normal University, Shanghai, 200241 China; 2 Collaborative Innovation Center of Extreme Optics, Shanxi University, Taiyuan, Shanxi 030006 China

## Abstract

An intermediate band in the band gap of semiconductors is fundamental to the development of the intermediate band solar cells, but it is usually produced artificially, which imposes technical challenges on the experimental realization. Here we found that there are natural intermediate bands in the band gaps of the I_2_-II-IV-VI_4_ quaternary chalcogenide semiconductors such as Cu_2_ZnSnS_4_ and Ag_2_ZnSnSe_4_, which had been proposed as promising light-absorber semiconductors in thin film solar cells. By first-principles calculations, we found the lowest conduction band of these I_2_-II-IV-VI_4_ semiconductors in the kesterite structure is isolated (a lone band, resulting from the energy separation between Sn 5s and 5p states), which can be viewed as a natural intermediate band. The gap between the intermediate band and higher-energy conduction band can be increased through changing the crystal structure from the zincblende-derived kesterite structure to the wurtzite-derived wurtzite-kesterite structure. In contrast, the intermediate-conduction band gap shrinks when the component element Sn is replaced by Ge (Cu_2_ZnGeS_4_), and the gap even disappears (intermediate band disappear) when Sn is replaced by Si (Cu_2_ZnSiS_4_). Through tuning the intermediate-conduction and intermediate-valence band gaps, we show that the wurtzite-kesterite structured Ag_2_ZnSnSe_4_ may be a potential light-absorber semiconductor in intermediate band solar cells.

## Introduction

The intermediate band solar cells have attracted intensive attention after Luque and Martí showed in 1997 that an energy-conversion efficiency as high as 63.2% can be achieved with a properly located intermediate band^1,2^. The efficiency is much higher than the theoretical efficiency limit at 33.7% of an ideal single-junction solar cell which is known as the Shockley-Queisser limit^3,4^.

In order to obtain a properly located intermediate band in a semiconductor, several methods have been proposed, such as using quantum nanostructures^5–11^, high concentration of impurities (forming impurity bands)^12–18^, highly mismatched alloys (HMAs)^19–24^ and so on^25,26^. However, the experimental realization of these ideas in practical devices is still very challenging technically. The quantum dots (QDs) have received intensive attention as the first practical intermediate band solar cell^1^, but the quantum dots solar cells (QDSC) are often plagued by marginal optical absorption^27^. Till now, the optical absorption of QDs is still much inferior to that of bulk materials^27,28^. The impurity-band method requires a very high concentration of doping, but it is usually challenging to dope such a high concentration of impurity elements into the crystalline semiconductors that can form an impurity band and meanwhile avoid forming impurity complexes and precipitates^29^. Forming intermediate bands in the highly mismatched alloys such as Zn_1−y_Mn_y_Te_x_O_1−x_ and GaN_x_As_1−x−y_P_y_ alloys opens up a window to the wide material space for searching possible semiconductors with intermediate bands^22,24^. In 2010, Lee and Wang even found that the highly lattice-mismatched alloys ZnTe_1−x_O_x_ with intermediate band states formed as a result of the coupling between the O impurity states can be used for intermediate band solar cells with an efficiency as high as 63%^20^. However, the synthesis or growth of these lattice-mismatched alloys stabilized by the configuration entropy is difficult and the control of the composition uniformity is especially challenging.

If one can design a stable semiconductor with an intermediate band naturally existing in its band gap, the current limit in the development of practical high-efficiency intermediate band solar cells may be overcome. In this paper, we found the I_2_-II-IV-VI_4_ series of quaternary chalcogenide semiconductors such as Cu_2_ZnSnS_4_, Cu_2_ZnGeS_4_, Ag_2_ZnSnS_4_ and Ag_2_ZnSnSe_4_ in the kesterite and wurtzite-kesterite crystal structures have natural intermediate bands according to the band structure calculation. Recently, these I_2_-II-IV-VI_4_ semiconductors have been studied both experimentally and theoretically as one of the most promising light-absorber materials to overtake Cu(In,Ga)Se_2_ (CIGS) in the commercialized CIGS thin film solar cells, because their component elements possess high abundance and less toxicity^30–33^. A record efficiency as high as 12.6% have been achieved in thin film solar cells based on their alloys Cu_2_ZnSn(S,Se)_4_^31^ which are very stable and composition-uniform and can be synthesized using different methods^30^.

Our calculations here showed that their lowest conduction band is an isolated band and can be viewed as a natural intermediate band, which results from the energy separation between the Sn 5s and 5p states. Furthermore, the gap between the intermediate band and higher-energy conduction band and the gap between the intermediate band and valence band can be tuned efficiently through changing the crystal structure or replacing the component elements, indicating that these I_2_-II-IV-VI_4_ semiconductors are very flexible for designing semiconductors with natural intermediate bands. Through tuning the intermediate-conduction and intermediate-valence band gaps to the optimal values according to the model of Luque and Martí, we show that the wurtzite-kesterite structured Ag_2_ZnSnSe_4_ may be a potential light-absorber semiconductor in intermediate band solar cells. These results provide a new strategy for the future development of the intermediate band solar cells.

## Results and Discussion

### Origin of the Intermediate Band

**Figure 1:**
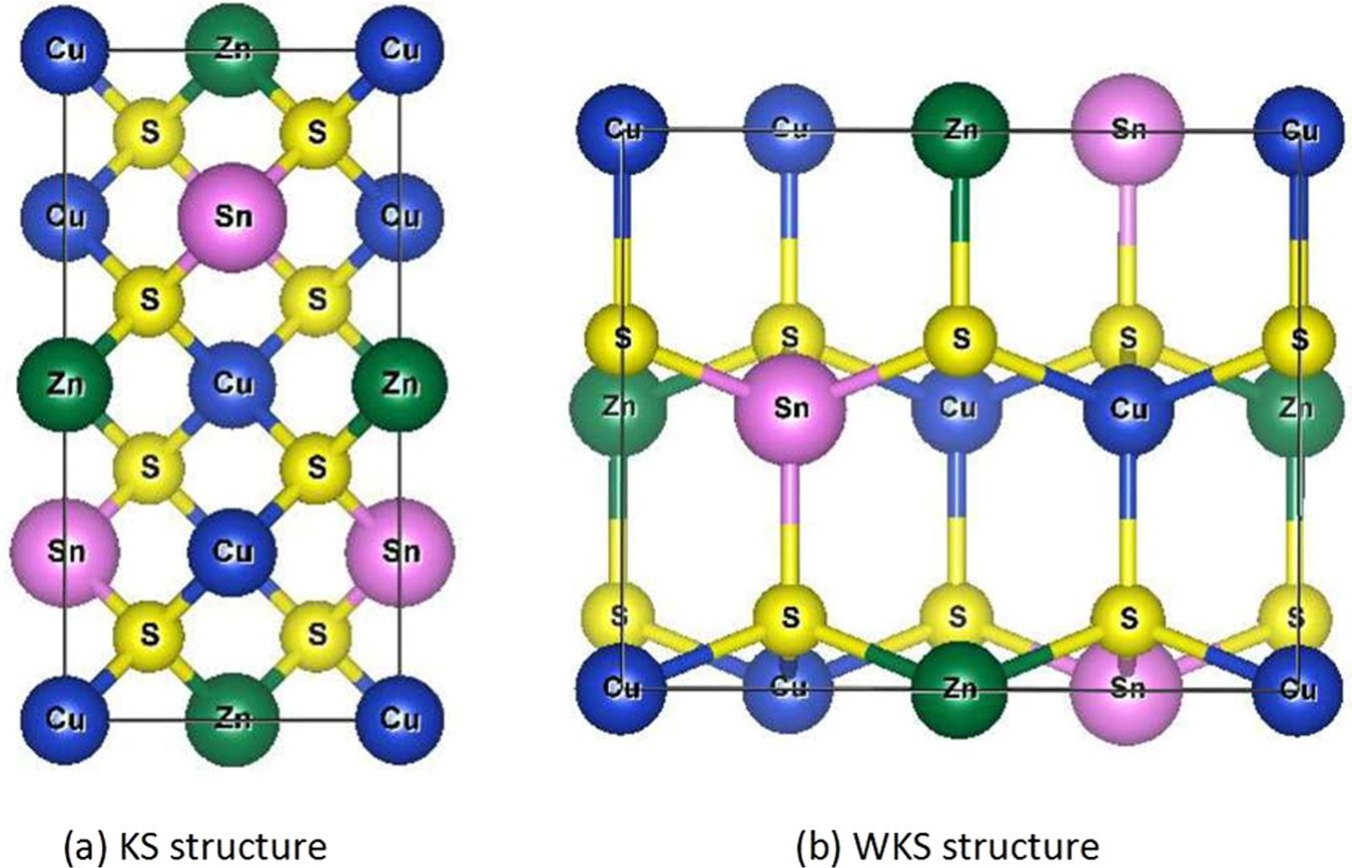
The (**a**) kesterite (KS) structure and (**b**) wurtzite-kesterite (WKS) structure of Cu_2_ZnSnS_4._

The crystal structure and electronic structure of the I_2_-II-IV-VI_4_ quaternary chalcogenide semiconductors have been intensively studied since 2008, because Cu_2_ZnSnS_4_ and Cu_2_ZnSnSe_4_ are candidate photovoltaic materials with high efficiency and earth-abundant component elements^34–38^. Five characteristic crystal structures derived from binary zincblende and wurtzite structures have been reported theoretically in literature^37,38^, among them the kesterite (KS)^37^ and wurtzite-kesterite (WKS)^38^ structures (as shown in Fig. 1) were predicted to have lower energy than other zincblende- and wurtzite-derived structures according to previous calculations^37,38^.

The band structures of kesterite structured Cu_2_ZnSnS_4_ have been calculated in the earlier studies^36,37,39^, which all showed that its lowest conduction band is isolated from the higher-energy conduction bands, i.e., the lowest conduction band is a lone band between the highest valence band and higher-energy conduction bands. Although this is clearly shown in the figures of the calculated band structure, the lone band did not attract special attention and most of the previous studies on Cu_2_ZnSnS_4_ and Cu_2_ZnSnSe_4_ are focused on their application as light absorber material in single-junction thin film solar cells.

**Figure 2:**
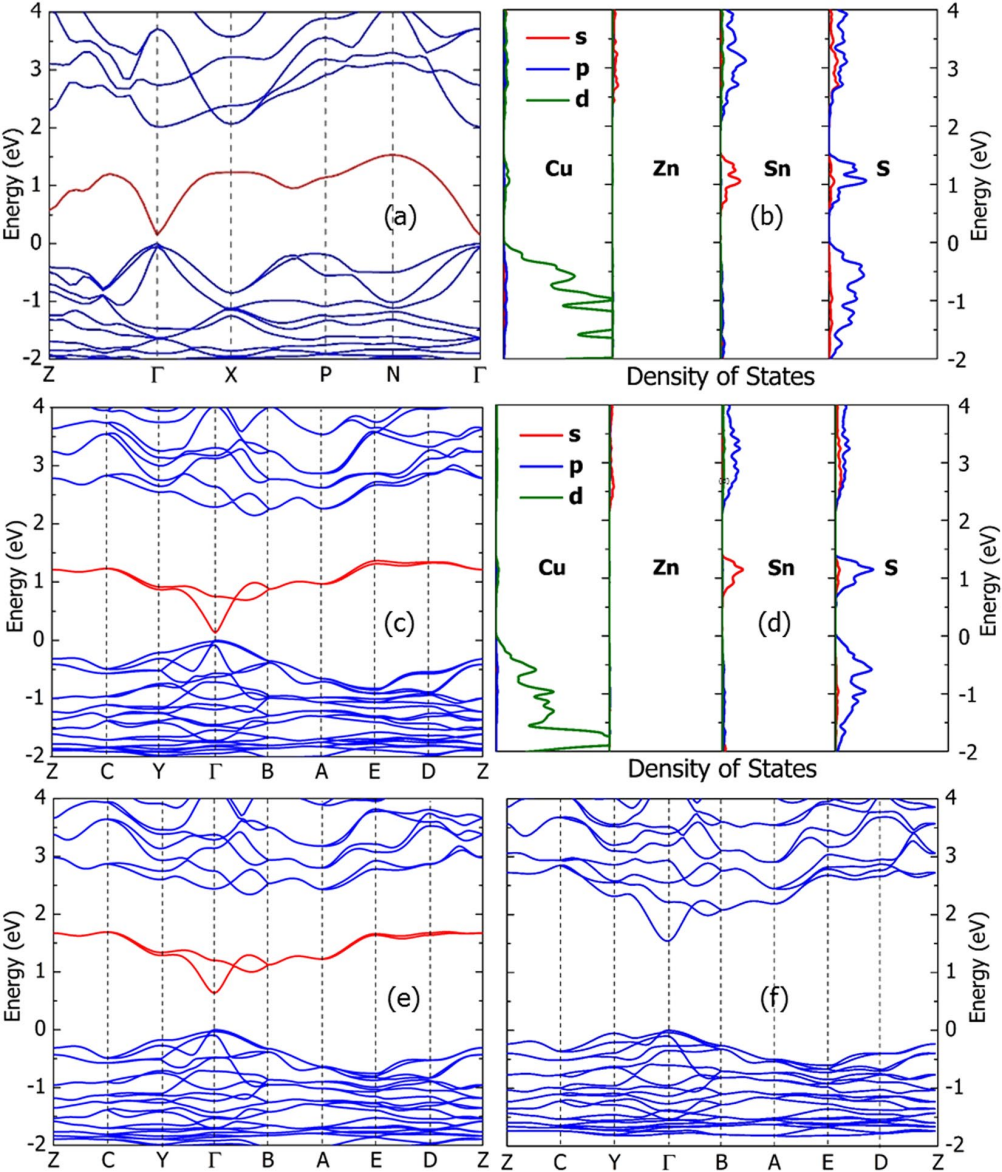
The calculated band structure of (**a**) kesterite structured Cu_2_ZnSnS_4_, (**c**) wurtzite-kesterite structured Cu_2_ZnSnS_4_, (**e**) wurtzite-kesterite structured Cu_2_ZnGeS_4_ and (**f**) wurtzite-kesterite structured Cu_2_ZnSiS_4_, (**b**) density of states projected on different elements in kesterite structured Cu_2_ZnSnS_4_ and (**d**) wurtzite-kesterite structured Cu_2_ZnSnS_4_. All the band structures are calculated using the GGA-PBE functional. The intermediate bands are represented by red lines. The high symmetry k-points in first Brillouin zone are along the following path: Z:(1/2,1/2,-1/2) → Γ:(0,0,0) → X:(0,0,1/2) → P:(1/4,1/4,1/4) → N:(0,1/2,0) → Γ:(0,0,0) for kesterite structured Cu_2_ZnSnS_4_ and Z:(0,1/2,0) → C:(1/2,1/2,0) → Y:(1/2,0,0) → Γ:(0,0,0) → B:(0,0,1/2) → A:(-1/2,0,1/2) → E:(-1/2,1/2,1/2) → D:(0,1/2,1/2) → Z:(0,1/2,0) for wurtzite-kesterite structured Cu_2_ZnSnS_4_, Cu_2_ZnGeS_4_ and Cu_2_ZnSiS_4_. The density of states of Cu_2_ZnGeS_4_ and Cu_2_ZnSiS_4_ is similar to that of Cu_2_ZnSnS_4_ as shown in (**d**), so it is not shown here.

In fact, this lone band can be considered as a natural intermediate band if it really has no connection with any other higher-energy conduction bands. To check whether the band is really isolated from other conduction bands, we performed the band structure calculations for both the kesterite and wurtzite-kesterite structured Cu_2_ZnSnS_4_ using the PBE functional. The calculated band structure and density of states of kesterite structured Cu_2_ZnSnS_4_ are shown in Fig. 2(a–b). Obviously, the lowest conduction band is really isolated from the higher-energy conduction bands in the whole Brillouin zone, and the lone band produces an isolated peak in the density of states.

To understand why the lone band is isolated from other higher-energy conduction bands, we calculated the partial density of states projected on the s, p, d orbitals of Cu, Zn, Sn and S, as shown in Fig. 2(b). The lone band is composed mainly of the Sn 5s orbital and S 3s and 3p orbitals, whereas the higher-energy conduction bands are composed mainly of the Sn 5p orbital and S 3s and 3p orbitals. The higher-energy conduction bands from I and II atoms (Such as Cu and Zn in Fig. 2) is negligible compared with IV and VI atoms (such as Sn and S in Fig. 2), so we will not discuss the influence of I and II atoms here. As we know, Sn takes the +4 charged state in Cu_2_ZnSnS_4_, so its 5s and 5p orbitals are unoccupied (with the electrons given to S) and contribute to the unoccupied conduction band states. Furthermore, the 5s and 5p states of Sn have hybridization with the lower-energy S 3s and 3p states, so the conduction bands are composed mainly of the anti-bonding states of the hybridization between Sn 5s, 5p and S 3s, 3p states. Since the Sn 5s states has lower energy than Sn 5p states, the lowest-energy conduction band is composed mainly of the antibonding state of the Sn 5s + S 3s, 3p hybridization, and the other higher-energy conduction bands are composed mainly of the antibonding states of the Sn 5p + S 3s, 3p hybridization. According to the band component analysis, we can understand the separation between the lowest-energy and higher-energy conduction bands.

**Figure 3:**
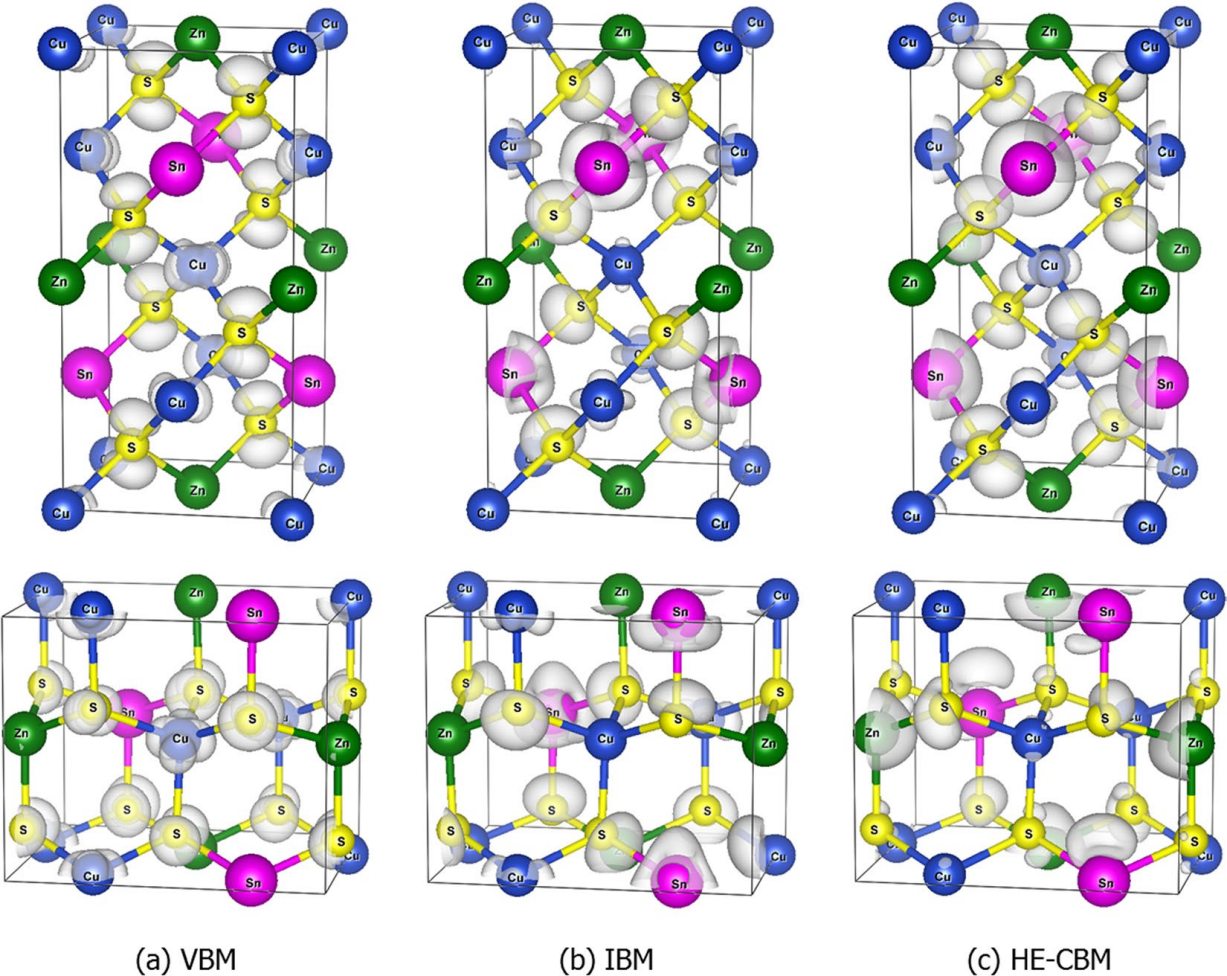
The norm square of the (**a**) valence band maximum (VBM) state, (**b**) the intermediate band minimum (IBM) state and (**c**) the higher-energy conduction band minimum (HE-CBM) state wave-function in the kesterite (top three figures) and wurtzite-kesterite (bottom three figures) structured Cu_2_ZnSnS_4_. The white contours show the isosurface of the norm squared wave-function with the isovalue at 0.003.

The analysis based on the calculated density of states is also supported by the plotted wave-function (shown in Fig. 3) of the valence band maximum (VBM), intermediate band minimum (IBM), and higher-energy conduction band minimum (HE-CBM) states at the Г point of the Brillouin zone for both kesterite and wurtzite-kesterite structured Cu_2_ZnSnS_4_. Obviously, the IBM state wave-function in Fig. 3(b) is localized on Sn (5s orbital) and S (3s orbital), and meanwhile there is no wave-function between the bonded Sn and S, indicating that the state is the antibonding state of the Sn -5s and S-3s hybridization. The HE-CBM state in Fig. 3(c) is also localized mainly on Sn (more like 5p states) and S (more like 3p states), and there is no wave-function between the bonded Sn and S either, indicating that the state is the antibonding state of the Sn-5p and S-3p hybridization. In contrast, the VBM state is localized mainly on Cu (Cu 3d states) and S (3p state), and it is the anti-bonding state of the Cu-S p-d hybridization.

### Tuning the Intermediate-Conduction Band Gap

Since the gap between the intermediate band (lowest-energy conduction band) and the higher-energy conduction band is 0.5 eV for the kesterite structured Cu_2_ZnSnS_4_, which is only suitable for the absorption of low-energy infrared photons. According to the model proposed by Luque and Martí^1,2^, the highest efficiency of intermediate band solar cells can be achieved with the intermediate-valence gap at about 1.2 eV and the intermediate-conduction gap at about 0.7 eV^1,2^. Therefore, it is necessary to tune the intermediate-conduction gap between the intermediate band and the higher-energy conduction band.

Here we propose two possible methods to tune the gap through changing the crystal structure of Cu_2_ZnSnS_4_, and changing the component element in Cu_2_ZnSnS_4_. Since the origin of the intermediate band in the kesterite structured Cu_2_ZnSnS_4_ is related to the Sn 5s and 5p states which hybridize with the S 3s and 3p states, similar intermediate band may also exist in Cu_2_ZnSnS_4_ crystallizing in other structures. In Fig. 2(c,d) the calculated band structure and density of states of the wurtzite-kesterite structured Cu_2_ZnSnS_4_ is shown. Obviously, there is also an intermediate band, and the gap between the intermediate band and the higher-energy conduction band is as large as 0.8 eV, larger than that for the kesterite structure. Therefore, it is possible to tune the gap through controlling the crystal structure of Cu_2_ZnSnS_4_. It should be noted that the intermediate band of the wurtzite-kesterite structured Cu_2_ZnSnS_4_ is actually composed of two bands, while that of the kesterite structured Cu_2_ZnSnS_4_ has only one band. This is due to the larger primitive cell of the wurtzite-kesterite structure which has two Sn atoms (therefore two lone bands composed of the two Sn 5s orbitals), than that of the kesterite structure which has only one Sn atom.

Through changing the component element in Cu_2_ZnSnS_4_, the gap between the intermediate band and higher-energy bands can also be tuned. Since the intermediate band is related to the element Sn, we expect that the gap can be tuned efficiently through replacing Sn by Ge or Si. As shown in Fig. 2(e), there is also an intermediate band in the calculated band structure of wurtzite-kesterite Cu_2_ZnGeS_4_, however, the gap between the intermediate band and higher-energy bands is only 0.7 eV, smaller than that of the wurtzite-kesterite Cu_2_ZnSnS_4_. That means the gap shrinks as the group IV element becomes smaller. Following this trend, the gap should become even smaller if we replace Ge by Si. Interestingly, the calculated band structure of Cu_2_ZnSiS_4_ in Fig. 2(f) shows that the intermediate band disappears, i.e., there is no gap between the intermediate band and higher-energy conduction band, and the lone band disappears. The shrinking of this intermediate-conduction band gap from Sn to Ge to Si should result from the stronger hybridization between Si and S than that between Ge and S (also between Sn and S), which make the s band and p bands merge after hybridizing with the S 3s and 3p bands strongly.

Since the intermediate-conduction band gap increases as the crystal structure is changed into the wurtzite-kesterite structure, and decreases as the component element Sn is replaced by Ge and Si, we will focus on the wurtzite-kesterite structure in the following discussion because a larger intermediate-conduction band gap is required for higher photovoltaic efficiency according to the theoretical model proposed by Luque and Martí^1,2^.

### Tuning the Intermediate-Valence Band Gap

The intermediate-valence band gap between the intermediate band (the lowest conduction band) and the valence band is actually the fundamental gap of these I_2_-II-IV-VI_4_ semiconductors. Previous experiments and calculations have shown that the fundamental gap of the kesterite or wurtzite-kesterite structured Cu_2_ZnSnS_4_ is about 1.5 eV^35,36^, slightly larger than the optimal intermediate-valence band gap according to the model of Luque and Martí^1,2^. Therefore, tuning the intermediate-valence band gap is also necessary, which can be achieved effectively through replacing Cu by Ag (shift the valence band downward), replacing Sn by Ge (shift the conduction band downward), or replacing S by Se (shift the conduction band downward and the valence band upward)^37^.

On the other hand, the intermediate band should be properly filled (the Fermi level is in the intermediate band) in a working intermediate band solar cell, so that the optical excitation of carriers from the intermediate band to the higher-energy conduction band can be strong enough to balance the optical excitation of carriers from the valence band to the intermediate band and a steady state can be maintained in the working cell. If the semiconductor is undoped and the intermediate band is fully unoccupied (the Fermi level is in the fundamental gap), the intermediate-conduction excitation will be weak. To dope the semiconductor properly, we have to consider the position of the valence and conduction band edge levels. Since the valence band edges of Cu_2_ZnSnS_4_ and other Cu-based I_2_-II-IV-VI_4_ semiconductors are high due to the shallow Cu 3d level, Cu_2_ZnSnS_4_ is intrinsically p-type, and the Fermi energy is limited to close to the valence band edge by the easy formation of intrinsic point defects^39^, so it is difficult to dope Cu_2_ZnSnS_4_ to n-type and shift the Fermi energy upward to close to the conduction band. As a result, the proper filling of the intermediate band by doping Cu_2_ZnSnS_4_ is impossible. If we replace Sn by Ge or replace S by Se, the existence of Cu in the compound still produces a high valence band and thus the proper filling (doping) of the intermediate band is still impossible. In contrast, replacing Cu by other elements such as Ag (with much lower 4d levels) may overcome the doping limit and shift the Fermi level to the intermediate band, which had been shown experimentally and theoretically^40,41^. However, it is still unknown how the Ag substitution of Cu influences the intermediate band, which will be discussed below.

**Figure 4:**
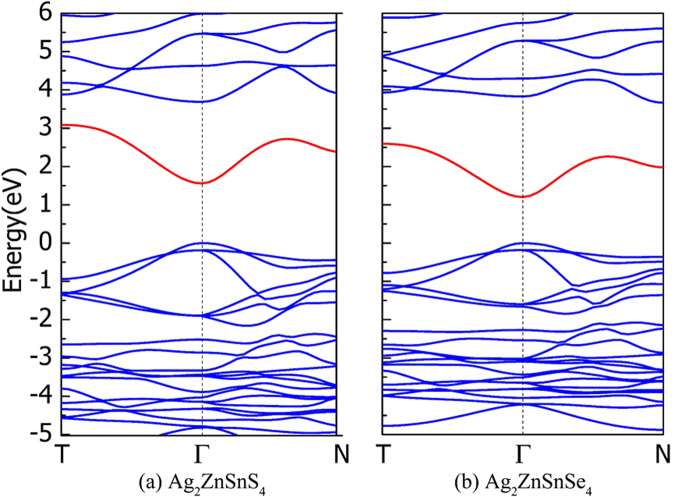
The calculated band structure of (**a**) kesterite structured Ag_2_ZnSnS_4_ and (**b**) kesterite structured Ag_2_ZnSnSe_4_, calculated using the HSE06 hybrid functional. The intermediate bands are represented by red lines. The high symmetry k-points in first Brillouin zone are along the following path: T:(0,0,1/2)→Γ:(0,0,0)→N:(1/2,1/2,1/2). Since the HSE06 functional is computationally expensive, so we considered only three representative high symmetry k-points here, less than those in Fig. 2.

In Fig. 4(a) we plot the calculated band structure of the kesterite-structured Ag_2_ZnSnS_4_. To avoid the serious band gap underestimation of the PBE functional, here the HSE06 hybrid functional is used to calculate the band structure (based on the crystal structure relaxed using PBE functional, which underestimates the band gap of Cu_2_ZnSnS_4_ by about 0.2 eV, *i.e*., the calculated value is about 1.3 eV and the experimental value is 1.5 eV). As we can see, there is still an intermediate band, indicating that the Ag_2_ZnSnS_4_ may be used in the intermediate band solar cells. However, the intermediate-valence band gap of the kesterite-structured Ag_2_ZnSnS_4_ is about 1.6 eV (the experimental band gap is 2.01 eV^42^), even larger than that of Cu_2_ZnSnS_4_ calculated using the same method (1.3 eV), so the simple replacement of Cu by Ag can overcome the doping, but does not lead to a smaller intermediate-valence band gap. In Fig. 4(b) we also plot the calculated band structure of kesterite-structured Ag_2_ZnSnSe_4_ (replace S by Se), in which the intermediate-valence band gap is decreased to about 1.2 eV (the experimental band gap is 1.34 eV^42^), smaller than that of Cu_2_ZnSnS_4_ (1.3 eV). Furthermore, the intermediate band also exists, indicating that Ag_2_ZnSnSe_4_ is a more suitable absorber semiconductor in intermediate band solar cells.

**Figure 5:**
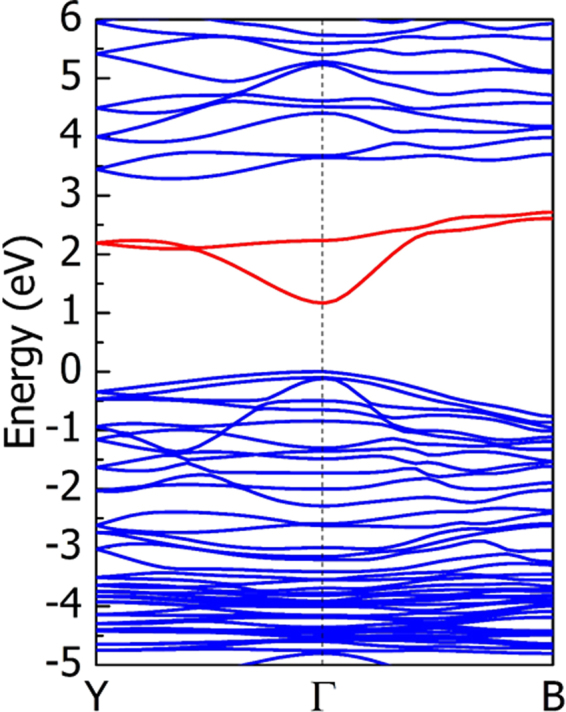
The calculated electronic band structure of wurtzite-kesterite structured Ag_2_ZnSnSe_4_, calculated using the HSE06 functional. The intermediate bands are represented by red lines. The high symmetry k-points in first Brillouin zone are along the following path: Y:(0,0,1/2)→Γ:(0,0,0)→B:(1/2,1/2,1/2). Since the HSE06 functional is computationally expensive, so we considered only three representative high symmetry k-points here, less than those in Fig. 2.

Although the intermediate-valence band gap is tuned to a smaller value and meanwhile the proper filling of the intermediate band is possible in Ag_2_ZnSnSe_4_, the intermediate-conduction band gap is unexpectedly increased to about 1 eV as shown in Fig. 4(b), which is higher than the optimal value according to the best performance model proposed by Luque and Martí^1,2^. Then, we should decrease this band gap. According to the discussion in the above section, the intermediate-conduction band gap may be decreased through changing the crystal structure from the kesterite structure to wurtzite-kesterite structure. In Fig. 5, we plot the calculated band structure of the wurtzite-kesterite structured Ag_2_ZnSnSe_4_, in which the intermediate-conduction band gap is decreased to about 0.7 eV, in accordance with our expectation, and the intermediate-valence band gap is still about 1.2 eV, close to that of the kesterite structure. Since both the intermediate-conduction and intermediate-valence band gaps can be tuned effectively, closer to the optimal values, we think that the wurtzite-kesterite structured Ag_2_ZnSnSe_4_ may be an ideal light-absorber semiconductor in the intermediate band solar cells.

It should be noted that the width of the intermediate band in Fig. 5 is about 1.2 eV, which is quite large and differ significantly from the infinitesimal bandwidth required by the model of Luque and Martí^1,2^. In 2008, Levy and Honsberg had considered both the positive effect of the finite band width (the recombination through the intermediate band can be suppressed) and the negative effect (the optical absorption is decreased), and showed that the optimal width of the intermediate band should be roughly equal to or lower than 825 meV^43^. Based on their study, we can find that the width of 1.2 eV of the intermediate band in wurtzite-kesterite structured Ag_2_ZnSnSe_4_ largely exceeds the optimal width. Therefore, the efficiency of the intermediate band solar cells based on the wurtzite-kesterite structured Ag_2_ZnSnSe_4_ may be limited to a low value by the finite band width which is similar to the case shown in Fig. 3 of their paper^43^. This may be an important problem in the development of practical solar cells. One possible way to solve this problem is to investigate other I_2_-II-IV-VI_4_ quaternary semiconductors and search for those with narrow intermediate band. On the other hand, although it is shown that the proper n-type doping and filling of the intermediate band is possible in Ag_2_ZnSnSe_4_, however, it is so far unknown which element is the optimal dopant that can have good solubility in this compound semiconductors and meanwhile produce high concentration of electron carriers filling the intermediate band. These problems deserve further study in the development of practical intermediate band solar cells based on the I_2_-II-IV-VI_4_ semiconductors.

## Conclusions

Using the density functional theory calculations, we found that the I_2_-II-IV-VI_4_ quaternary chalcogenide semiconductors, Cu_2_ZnSnS_4_, Cu_2_ZnGeS_4_, Ag_2_ZnSnS_4_ and Ag_2_ZnSnSe_4_, have natural intermediate bands in their band gaps. These semiconductors have already been synthesized and their intermediate bands are intrinsic in their band gaps, which is an important advantage compared to the intermediate bands introduced artificially by extrinsic doping or forming highly lattice-mismatched alloys. The intermediate-conduction and intermediate-valence band gaps can be tuned efficiently through changing the crystal structure or the component elements in this quaternary compound semiconductors. The wurtzite-kesterite structured Ag_2_ZnSnSe_4_ have the band gaps close to the optimal band gaps according to the model of Luque and Martí, so it may be an ideal light-absorber semiconductor in intermediate band solar cells. However, the width of the intermediate band of Ag_2_ZnSnSe_4_ is relatively large, which may limit the efficiency, and a proper doping is also required to shift the Fermi energy to the intermediate band. These problems deserve further study in the development of practical intermediate band solar cells based on the I_2_-II-IV-VI_4_ semiconductors. We call for experimental investigation on these semiconductors with natural intermediate bands.

### Calculation Methods

All the total energy and band structure calculations were performed using the density functional theory methods as implemented in the Vienna Ab initio simulation package (VASP) code^44^. For the exchange-correlation potential, we used the generalized gradient approximation (GGA)^45^ of Perdew-Burke-Ernzerhof (known as PBE)^46^ to relax the crystal structures and calculate the band structure of Cu_2_ZnSnS_4_, Cu_2_ZnGeS_4_, and Cu_2_ZnSiS_4_. Since PBE is known to underestimate the s-p band gap of semiconductors significantly, which affects our investigation on the intermediate-valence band gap (which is a s-p band gap), the Heyd-Scuseria-Ernzerhof (HSE06) hybrid functional^47,48^ is also used to calculate the band structure. In Fig. 2 the band structures are calculated using the PBE functional for a series of high-symmetry k-points, from which we can know the shape of the band structure and determine the k-points where the band gaps open. In Fig. 4 and Fig. 5 the band structures are calculated using the computationally heavier HSE06 functional, so we consider only three representative high-symmetry k-points, from which we can determine the band gap size accurately.

The cutoff of the kinetic energy for the plane-wave basis wave functions is chosen to be 400 eV for all the calculations. For the Brillouin zone integration, we used the 4 × 4 × 4 Monkhorst-Pack k-point meshes^49^ for the wurtzite-kesterite structure and equivalent k-point meshes for the kesterite structure. All the lattice parameters and atomic positions were fully relaxed until the quantum mechanical force on each atom is smaller than 0.01 eV/Å. Convergence test calculations showed that the larger energy cutoff, denser k-point meshes and smaller force criterion changes the calculated band gap by less than 0.01 eV.
